# Pan-cancer analysis of oncogenic *TNFAIP2* identifying its prognostic value and immunological function in acute myeloid leukemia

**DOI:** 10.1186/s12885-022-10155-9

**Published:** 2022-10-15

**Authors:** Mei-si Lin, Hui-Yun Zhong, Rita Lok-Hay Yim, Qi-Yan Chen, Hong-ling Du, Hao-qi He, Ke Lin, Peng Zhao, Ru Gao, Fei Gao, Min-Yue Zhang

**Affiliations:** 1 State Key Laboratory of Southwestern Chinese Medicine Resources, Pharmacy School, Chengdu University of Traditional Chinese Medicine, Chengdu, 611730 China; 2 Sichuan Vocational College of Health and Rehabilitation, Zigong, 643000 China; 3 Department of Medicine, Queen Mary Hospital, The University of Hong Kong, Pokfulam, Hong Kong, SAR, China; 4 School of Medical Information Engineering, Chengdu University of Traditional Chinese Medicine, Chengdu, 611730 China; 5 Department of Nursing, Chengdu Wenjiang People’s Hospital, Chengdu, 611100 Sichuan China; 6 Division of Hematology, Renji Hospital, School of Medicine, Shanghai Jiaotong University, Shanghai, 200127 China

**Keywords:** Acute myeloid leukemia, *TNFAIP2*, Prognosis, Immunomodulation, Oncogenic

## Abstract

**Background:**

Tumor necrosis factor alpha-induced protein 2 (*TNFAIP2*), a TNFα-inducible gene, appears to participate in inflammation, immune response, hematopoiesis, and carcinogenesis. However, the potential role of *TNFAIP2* in the development of acute myeloid leukemia (AML) remains unknow yet. Therefore, we aimed to study the biological role of *TNFAIP2* in leukemogenesis.

**Methods:**

*TNFAIP2* mRNA level, prognostic value, co-expressed genes, differentially expressed genes, DNA methylation, and functional enrichment analysis in AML patients were explored via multiple public databases, including UALCAN, GTEx portal, Timer 2.0, LinkedOmics, SMART, MethSurv, Metascape, GSEA and String databases. Data from The Cancer Genome Atlas (TCGA), Gene Expression Omnibus (GEO) and Beat AML database were used to determine the associations between *TNFAIP2* expression and various clinical or genetic parameters of AML patients. Moreover, the biological functions of *TNFAIP2* in AML were investigated through in vitro experiments.

**Results:**

By large-scale data mining, our study indicated that *TNFAIP2* was differentially expressed across different normal and tumor tissues. *TNFAIP2* expression was significantly increased in AML, particularly in French–American–British (FAB) classification M4/M5 patients, compared with corresponding control tissues. Overexpression of *TNFAIP2* was an independent poor prognostic factor of overall survival (OS) and was associated with unfavorable cytogenetic risk and gene mutations in AML patients. DNA hypermethylation of *TNFAIP2* at gene body linked to upregulation of *TNFAIP2* and inferior OS in AML. Functional enrichment analysis indicated immunomodulation function and inflammation response of *TNFAIP2* in leukemogenesis. Finally, the suppression of *TNFAIP* resulted in inhibition of proliferation by altering cell-cycle progression and increase of cell death by promoting early and late apoptosis in THP-1 and U937AML cells.

**Conclusion:**

Collectively, the oncogenic *TNFAIP2* can function as a novel biomarker and prognostic factor in AML patients. The immunoregulation function of *TNFAIP2* warrants further validation in AML.

**Supplementary Information:**

The online version contains supplementary material available at 10.1186/s12885-022-10155-9.

## Introduction

Acute myeloid leukemia (AML) is characterized by a group of genetically heterogeneous hematological malignancies which are caused by the malignant transformation and clonal expansion of myeloid progenitor cells in the bone marrow. The incidence of AML increases with age and the median age at diagnosis is about 70 years old [1]. The clinical prognosis of AML varies widely, depending on the genetic and epigenetic alterations detected [2]. In recent years, there has been arising significance of tumor immunology in the pathogenesis of AML with increasing evidence of favorable outcomes from immune inhibitory molecules and leukemic antigens [3–7]. Theretofore, clinical application of immuno-oncology therapy is expected to become an alternative regime to conventional chemotherapy, with or without hematopoietic stem cell transplantation [3, 8–10].

Tumor necrosis factor alpha-induced proteins (TNFAIPs) is a protein family which can be induced by proinflammatory cytokine TNF-α. TNFAIPs family consists of eight members: TNFAIP1, TNFAIP2, TNFAIP3, TNFAIP4 (EFNA1), TNFAIP5 (PTX3), TNFAIP6 (TSG6), TNFAIP8 and TNFAIP9 (STEAP4) [11]. Previous studies suggested that TNFAIPs commonly played key roles in carcinogenesis, immune response, and inflammation [11–16]. These proteins primarily respond to TNF-α while TNFAIPs’ family members share less than 15% amino acid homology [11], suggesting that each member putatively have different biological functions.

*TNFAIP2*, also called B94 or M-Sec, is localized to 14q32.32 and codes a protein containing 654 amino acids. In addition to TNF-α, *TNFAIP2* expression can be also activated by other cytokines, such as IL-lβ, lipopolysaccharide, interferon-γ [17, 18]. *TNFAIP2*, which is found to be enriched in the spleen, male mature germ cells, hematopoietic and lymphoid tissues [11], participates in various physiological processes, including inflammation, angiogenesis, cell differentiation, proliferation [17, 19, 20]. Dysregulation of *TNFAIP2* and its oncogenic role have been reported in a variety of cancers [11, 16, 21–25]. However, the understanding of *TNFAIP2* in hematological disorders such as AML is very scarce. Due to the lack of knowledge of *TNFAIP2* in leukemogenesis, we herein carried out an in-depth study on *TNFAIP2* expression and its prognostic value in a range of cancers. Furthermore, the clinical significance, potential molecular functions, and regulatory networks of *TNFAIP2* in AML patients will be studied by bioinformatics analysis of datasets available on public databases.

## Materials and methods

### Downloading and processing data from public databases

The Cancer Genome Atlas (TCGA) gene expression RNA-seq data (containing 9359 TCGA tumor tissues, 727 TCGA normal tissues from 33 types of cancer) and The Genotype-Tissue Expression (GTEx) gene expression RNA-seq data of 31 normal tissues were downloaded using UCSC Xena (https://xena.ucsc.edu/). Toil software was applied to processes raw RNA-seq data and extract *TNFAIP2* gene expression data from across GTEx and TCGA datasets for subsequent analyses [26]. Gene expression profile of GSE14468 dataset from Gene Expression Omnibus (GEO) database (http://www.ncbi.nlm.nih.gov/geo/), containing RNA-seq data of 461 AML patients [27], as well as Beat AML dataset (http://www.vizome.org) [28] were also used to investigate the relations between *TNFAIP2* mRNA expression and clinicopathological factors of AML patients.

### Analysis of *TNFAIP2* expression in tumor and normal tissues

The UALCAN database (http://ualcan.path.uab.edu/), containing RNA-seq and clinical data of 33 cancer types from TCGA dataset [29], was used for the analysis of *TNFAIP2* expression in different types of tumor samples. The box plots were downloaded from the UALCAN website.

The GTEx portal (https://www.gtexportal.org/home/), containing RNA-seq data from 54 non-diseased tissues sites across nearly 1000 individuals, was used for the analysis of *TNFAIP2* expression in different normal tissues. The box plots were downloaded from the GTEx portal website.

To investigate the differential expression of *TNFAIP2* between tumors and normal tissues across different types of cancers from GTEx and TCGA datasets, two-sample Student’s t-test was applied if the data in each group were normally distributed; otherwise, the Mann–Whitney U test was applied. RNA-seq data were normalized by Log2 transformation. IBM SPSS Statistics 25 software was used to analyze the data.

### *TNFAIP2* analysis in cell lines available from Cancer cell line encyclopedia (CCLE) database

CCLE database (https://portals.broadinstitute.org/ccle), which provides RNA-seq data, DNA methylation data, gene mutation and copy number data of 1457 human cancer cell lines [30], were used to compare *TNFAIP2* expression levels among different cancer cell lines. The box plots were downloaded from the CCLE website.

### Survival analysis

Timer 2.0 (http://timer.comp-genomics.org) [31] was usually applied to explore the prognostic significance of genes in different types of cancers. We explored the prognostic values of *TNFAIP2* expression for overall survivals in pan-cancers by using these two databases. Kaplan-Meier survival analysis and the log-rank test were conducted to calculate *P*-value.

### Genetic alteration analysis

Pan-cancer analysis of *TNFAIP2* genetic alterations of were performed with cBioPortal web (https://www.cbioportal.org/) according to online instructions [32, 33]. The results of genetic alteration characteristics of *TNFAIP2*, including genetic alteration frequency, mutation type and CNA (copy number alteration) among different tumors from TCGA database were shown in the “Cancer Types Summary” module of cBioPortal web.

### *TNFAIP2* DNA methylation analysis

Two public databases, Shiny Methylation Analysis Resource Tool (SMART) App (http://www.bioinfo-zs.com/smartapp/) database [34] and MethSurv database (https://biit.cs.ut.ee/methsurv/) [35], which containing Infinium Human Methylation 450 K BeadChip data, RNA-seq data and clinical data of 33 cancer types from TCGA dataset, were employed to analyze *TNFAIP2* DNA methylation level in AML patients. The associations between *TNFAIP2* DNA methylation level and its expression as well as prognostic value on AML patients’ OS were explored.

### Co-expression genes and differentially expressed genes (DEGs) analysis

LinkedOmics database (http://www.linkedomics.orglogin.php) [36] was applied to determine the co-expressed genes correlated with *TNFAIP2* expression in the RNA-seq data of AML patients from the TCGA cohort. The Pearson correlation coefficient was calculated, and the volcano map of the co-expressed genes was plotted from the LinkedOmics website. The results of co-expression with *TNFAIP2* and immune-related genes were presented as heatmap, generated using the Limma package in R 3.6.3.

The AML patients from TCGA dataset were divided into two groups (*TNFAIP2* ^low^ and *TNFAIP2* ^high^) according to the median values of *TNFAIP2* mRNA from RNAseq data. The Limma package in R 3.6.3 was used to screen and plot volcano map of DEGs between the *TNFAIP2* ^low^ and *TNFAIP2* ^high^ groups of AML patients. Then Draw Venn diagrams online tool (http://bioinformatics.psb.ugent.be/webtools/Venn/) was applied to explore the overlapping genes between DEGs and co-expressed genes for further enrichment analysis.

### Functional enrichment and protein-protein interaction (PPI) analysis

Functional enrichment analyses of screened overlapping genes, including Gene Ontology (GO), Kyoto Encyclopedia of Genes and Genomes (KEGG) pathways and tissues enrichment analysis, were performed by using Metascape database (http://metascape.org/gp/index.html#/main/step1) [37].

Gene Set Enrichment Analyses (GSEA) of screened overlapping genes were performed by GSEA v4.1.0 database (www.broadinstitute.org/gsea) to identify AML-related enriched signaling pathways [38, 39]. We selected “c2.cp.kegg.v7.1.symbols.gmt” from MSigDB gene set as reference gene set when performing all GSEA analyses. For statistical analyses of enriched signaling pathways, normalized *P* <  0.05, false discovery rate (FDR) q <  0.25 and normalized enrichment score (NES) > 1.5 was considered as statistical significance.

The String database (https://string-db.org/) was employed to conduct PPI network analysis [40]. Visualization of PPI network and identification of hub genes among the PPI network were performed by software Cytoscape_v3.6.1. plugin MCODE [41].

### *TNFAIP2* knockdown

The *TNFAIP2* shRNA and scrambled control shRNA were inserted into the hU6-MCS-CMV-Puromycin (GV112) lentiviral vector. The target sequence of *TNFAIP2* for knockdown was listed below: GGATGTCCATGGAGCAGAATT. ShRNA lentivirus was applied to generate stable *TNFAIP2*-knockdown cells. The *TNFAIP2*-containing construct and packaging plasmid (Helper 1.0 and Helper 2.0 plasmids) were mixed and then co-transfected into 239 T cells. Viral supernatants were collected 48 h post-transfection. The viral particles were concentrated, and aliquots were stored at − 80 °C. Viral titers of concentrated particles were 4.5 × 10^8^ TU/ml. AML cell lines THP-1 and U937 cells were seeded in 12-well culture plates at the density of 8*10^4^ cells/well and maintained in RPMI with 10% FBS for 24 hours prior to transfection. The viral particles were added into THP-1 and U937 cells and continued to culture for 12 hours. Then the supernatant was removed by centrifuge and the transfected cells were cultured in RPMI with 10% FBS for further experiments.

### Quantitative reverse transcription polymerase chain reaction (qRT-PCR)

Total RNA was isolated with the SuperfecTRI, Total RNA Isolation Reagent kit (Shanghai Pufei Biotechnology), followed by reverse transcription with M-MLV Reverse Transcriptase (Promega). qRT-PCR was performed with SYBR Master Mixture (TAKARA), and the human Actin Beta *(ACTB)* was used as the endogenous control. The primer sequences for *TNFAIP2* and *ACTB* were listed as follows, *TNFAIP2* forward primer: 5′-GGCCAATGTGAGGGAGTTGAT-3′, reverse primer: 5′-CCCGCTTTATCTGTGAGCC-3′; *ACTB* forward primer: 5′-GCGTGACATTAA GGAGAAGC-3′, reverse primer: 5′-CCACGTCACACTTCATGATGG-3′. The relative quantity of *TNFAIP2* expression was calculated by the method of 2-^ΔΔCt^ and normalized against the endogenous control.

### CCK-8 assay, cell-cycle assay and Annexin V-APC & PI assay

The cell growth was analyzed using CCK-8 assay. Briefly, THP-1 or U937 cells from each stably transduced samples were seeded in 96-well plates (2*10^4^ cells/well) in 100 μl of medium and pre-incubated at 37 °C, 5% CO_2_, in a humidified atmosphere for 0, 24, 48, 72 or 96 hours. Counting Kit-8 kit (CCK-8) solution (10 μL, Dojindo Molecular Technologies, Kyushu, Japan) was then added to each well and the plate was incubated for 4 hours at 37 °C, 5% CO2, in a humidified atmosphere, followed by the measurement of absorbance at 450 nm using a microplate reader.

For cell-cycle assay, THP-1 or U937 cells (2*10^6^ cells) transfected with *TNFAIP2* shRNA or scramble control for 120 h were collected and washed three times with cold PBS. Cells were then incubated in 50 μg/ml PI staining solution with 100 μg/ml RNase A for 30 min at 4 °C for at least 2 hours, followed by the analyze of flow cytometry (BD Biosciences, USA).

Cell apoptosis was measured by Annexin V-PI assay (eBioscience). Briefly, THP-1 or U937 cells (5*10^5^ cells) transfected with *TNFAIP2* shRNA or scramble control for 120 hours were harvested, washed with cold PBS, and resuspended in 200 μl of Binding Buffer. Then 10 μl of Annexin-V-APC and 5 μl of PI were added to samples. The cells were incubated for 15 mins at room temperature in dark. Lastly, 800 μl of binding buffer were added to each sample. Samples were analyzed by flow cytometry (BD Biosciences, USA). Apoptotic cells included cells in early apoptosis phase (Annexin V positive, PI negative) and late apoptosis phase (Annexin V positive, PI positive).

Each assay was performed in triplicates of each sample in three independent experiments. Data were plotted by mean ± standard deviation.

### Statistical analysis

IBM SPSS 25.0. were used to conduct statistical analysis. χ^2^ test were applied to compare the correlation between *TNFAIP2* expression and clinicopathological parameters, including French–American–British (FAB) classification, sex, cytogenetic risk, and chromosome alterations. Student T test or ANOVA test were used to analyze the difference of continuous variables, including age, white blood cells count, the percentage of blast cells in bone marrow or peripheral blood, *TNFAIP2* mRNA levels, CCK-8 assay, cell cycle assay and Annexin V-APC & PI assay among different groups if the values in each group are normally distributed. Otherwise, the Manny-Whitney U test or Kruskal–Wallis test was used. All *P*-values were 2-sided and *P* <  0.05 was considered as statistical significance.

## Results

### Tissue-specific expression patten of *TNFAIP2* in pan-cancer

**Fig. 1:**
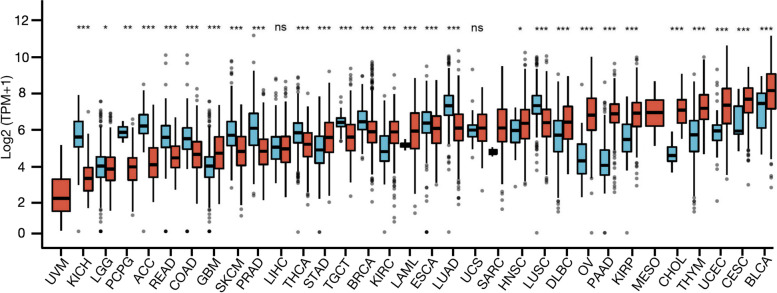
Differential expression of *TNFAIP2* in cancer tissues and normal counterparts from TCGA and GTEx databases, analyzed by Mann–Whitney U test. *P <  0.05, **P < 0.01, ***P < 0.001. (LAML, acute myeloid leukemia; ACC, adrenalcortical carcinoma; BLCA, bladder urothelial carcinoma; BRCA, breast invasive carcinoma; CHOL, cholangiocarcinoma; COAD, colon adenocarcinoma; DLBC, diffuse large B-cell lymphoma; ESCA, esophageal carcinoma; GBM, glioblastoma multiforme; HNSC, Head and Neck squamous cell carcinoma; KICH, kidney chromophobe; KIRC, kidney renal clear cell carcinoma; KIRP, kidney renal papillary cell carcinoma; LGG, brain lower grade glioma; LIHC, liver hepatocellular carcinoma; LUAD, lung adenocarcinoma; LUSC, lung squamous cell carcinoma; MESO, mesothelioma; OV, ovarian serous cystadenocarcinoma; PAAD, pancreatic adenocarcinoma; PCPG, pheochromocytoma and paraganglioma; PRAD, prostate adenocarcinoma; READ, rectum adenocarcinoma; SARC, sarcoma; SKCM, skin cutaneous melanoma; STAD, stomach adenocarcinoma; TGCT, testicular germ cell tumor; THCA, thyroid carcinoma; THYM, thymoma; UCEC, uterine corpus endometrial carcinoma; UVM, uveal melanoma)

The physiologic *TNFAIP2* mRNA levels across different normal tissues were first analyzed by using GTEx dataset. As shown in Supplementary Fig. S1a, the expression levels of *TNFAIP2* were highest in lung, spleen, and adipose tissues while lowest in muscle, pancreas, and brain tissues. The expression level in whole blood was intermediate among all the normal tissues. In the TCGA data, *TNFAIP2* mRNA levels in 33 types of tumor tissues were also explored (Fig. 1) and all cancers expressed *TNFAIP2.* The highest *TNFAIP2* level was observed in bladder urothelial carcinoma (BLCA), cervical squamous cell carcinoma and endocervical adenocarcinoma (CESC) and uterine corpus endometrial carcinoma (UCEC). The lowest *TNFAIP2* expression was observed in brain lower grade glioma (LGG), kidney chromophobe (KICH) and uveal melanoma (UVM), and intermediate expression was observed in AML. Additionally, *TNFAIP2* mRNA expression in 1457 cell lines derived from 26 tumor types in the CCLE database were also analyzed (Supplementary Fig. S1b). The results showed that cell lines from upper-aerodigestive tract and pancreas were the top two cell lines expressing the highest levels of *TNFAIP2* mRNA. Cell lines from lymphatic system (e.g., lymphoma and myeloma) expressed relatively less *TNFAIP* while intermediate expression level of *TNFAIP2* was observed in AML cell lines.

Subsequently, *TNFAIP2* expression levels between cancer and normal samples from 33 cancers were compared against each other in both TCGA and GTEx dataset (Fig. 1). Except for those cancers [mesothelioma (MESO), SARC (sarcoma) and UVM] whose normal tissue data was unavailable or too few, significant differences in *TNFAIP2* expression between tumor and normal tissue were found in 28 types of cancer. Among them, *TNFAIP2* was up-regulated in head and neck squamous cell carcinoma (HNSC), stomach adenocarcinoma (STAD), diffuse large B-cell lymphoma (DLBCL), glioblastoma multiforme (GBM), BLCA, AML, kidney renal clear cell carcinoma (KIRC), UCEC, kidney renal papillary cell carcinoma (KIRP), thymoma (THYM), CESC, cholangial carcinoma (CHOL), ovarian serous cystadenocarcinoma (OV)， and pancreatic adenocarcinoma (PAAD) compared with corresponding normal counterpart. In contrast, *TNFAIP2* were downregulated in KICH, adrenocortical carcinoma (ACC), pheochromocytoma and paraganglioma (PCPG), prostate adenocarcinoma (PRAD), lung adenocarcinoma (LUAD), rectum adenocarcinoma (READ), lung squamous cell carcinoma (LUSC), skin cutaneous melanoma (SKCM), colon adenocarcinoma (COAD), testicular germ cell tumor (TGCT), thyroid carcinoma (THCA), breast invasive carcinoma (BRCA), esophageal carcinoma (ESCA) and LGG. However, there was no significant difference of *TNFAIP2* mRNA levels in uterine carcinosarcoma (UCS) or liver hepatocellular carcinoma (LIHC) compared with their normal counterpart.

### Genetic and epigenetic alterations of *TNFAIP2* in AML

**Fig. 2:**
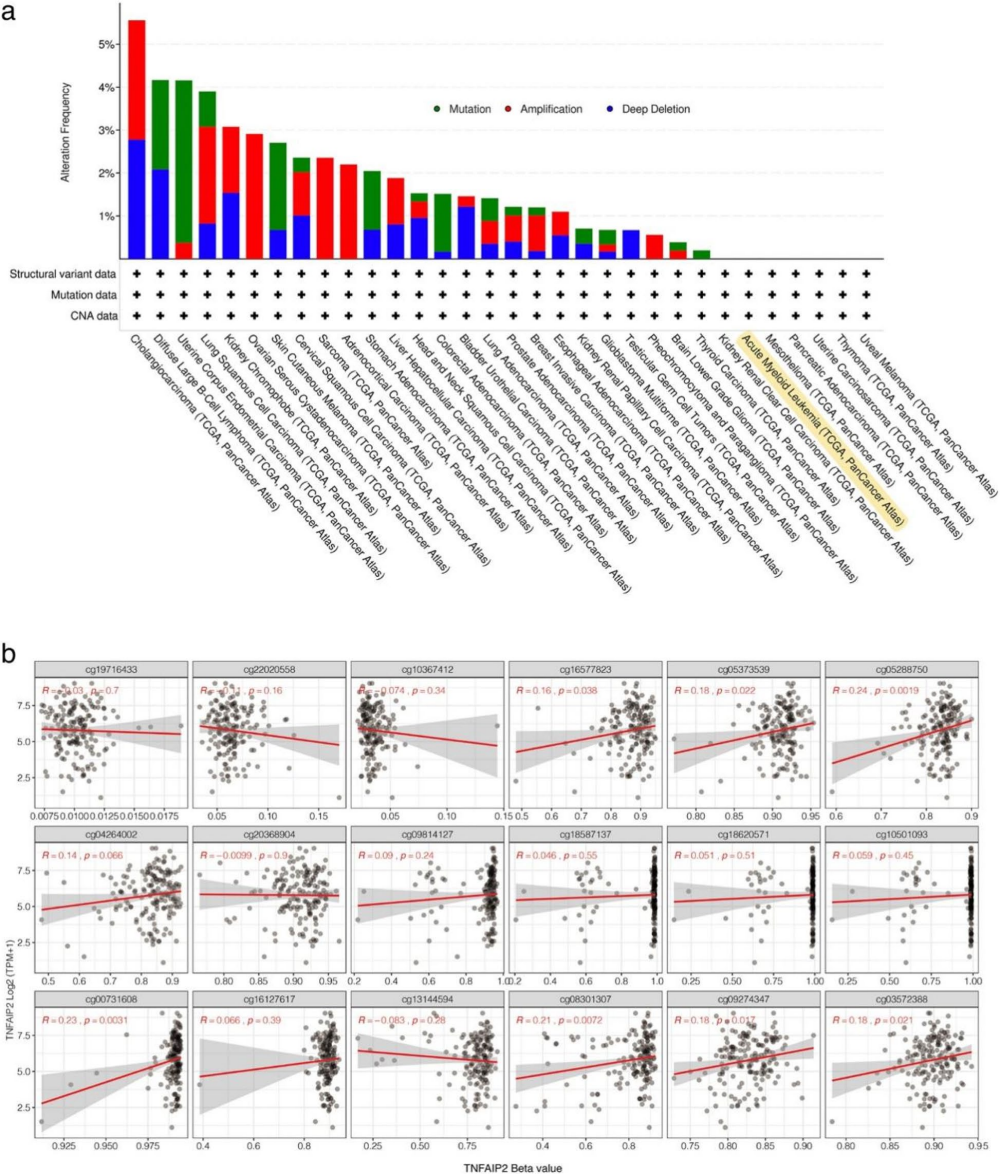
Genetic and epigenetic alterations of *TNFAIP2* in AML patients from TCGA dataset. **a** Genetic features of *TNFAIP2*, including mutation, amplification, and deletion, in different tumors of TCGA analyzed by cBioPortal. **b** Correlation of *TNFAIP2* methylation level and gene expression in AML patients analyzed by SMART App

Then the genetic alteration status of *TNFAIP2* gene in various types of cancer from TCGA cohorts was explored on cBioPortal web. As shown in Fig. 2a, the highest alteration frequency of *TNFAIP2* (5.56%) was observed in CHOL patients with “amplification” and “deep deletion” as the primary alterations. Nevertheless, none of AML cases had genetic alterations of *TNFAIP2* gene.

We subsequently used SMART App and MethSurv database to study *TNFAIP2* DNA methylation status in AML patients from the TCGA dataset. With respect to the probes at the promoter region of *TNFAIP2* gene (cg19716433, cg22020558 and cg10367412), there was no correlation between *TNFAIP2* DNA methylation level (β-value) and *TNFAIP2* expression level. However, we observed that hypermethylation of *TNFAIP2* at gene body region (cg16577823, cg05373539, cg05288750, cg00731608, cg08301307, cg09274347 and cg03572388) was positively correlated with gene expression of *TNFAIP2* (Fig. 2b and Supplementary Fig. S2a). Furthermore, Kaplan-Meier survival analysis suggested that AML patients with high methylation level of *TNFAIP2* at gene body (cg00731608, cg13144594 and cg08301307) exhibited inferior overall survival (OS) (Supplementary Fig. S2b).

### Multifaceted prognostic value of *TNFAIP2* expression across cancers

**Fig. 3:**
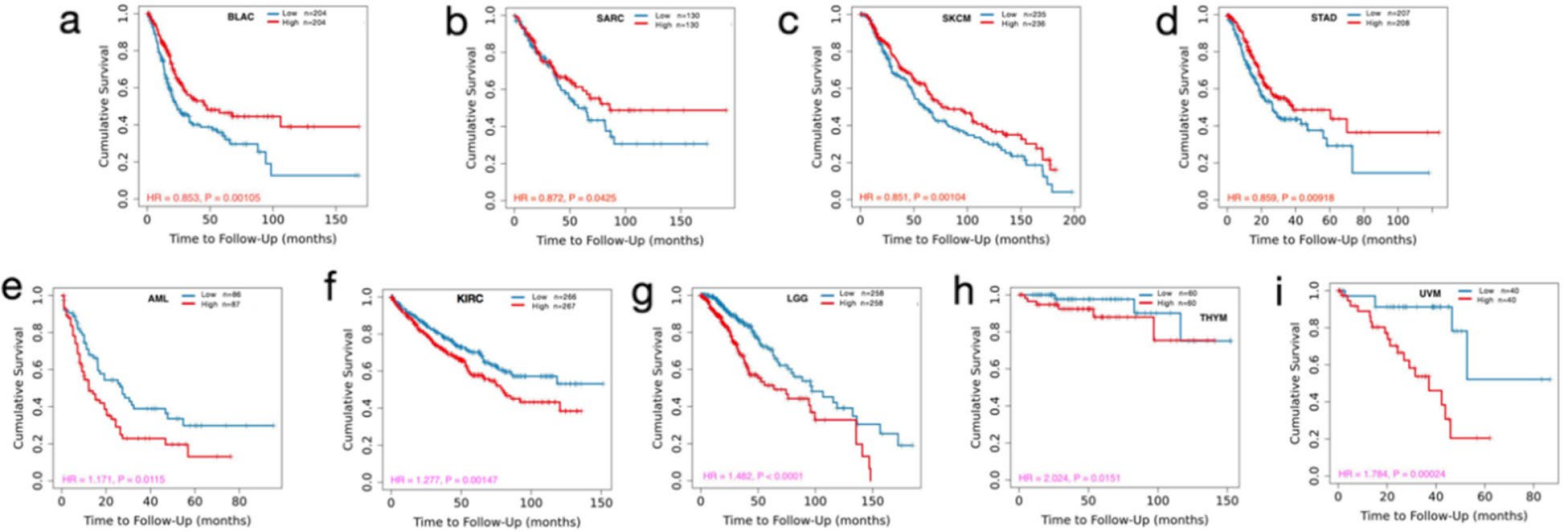
Analysis of the prognostic value of *TNFAIP2* expression on overall survival (OS) in different types of cancers by Timer 2.0 database. **a** BLAC, (**b**) SARC, (**c**) SKCM, (**d**) STAD, (**e**) LAML, (**f**) KIRC, (**g**) LGG, (**h**) THYM, (**i**) UVM. (LAML, acute myeloid leukemia; BLCA, bladder urothelial carcinoma; KIRP, kidney renal papillary cell carcinoma; LGG, brain lower grade glioma; SARC, sarcoma; SKCM, skin cutaneous melanoma; STAD, stomach adenocarcinoma; THYM, thymoma; UVM, uveal melanoma)

Upon basic evaluation of *TNFAIP2* expression across different tumors, *TNFAIP2* expression association with OS of cancer patients were studied. By using Timer 2.0 database which mainly based on RNA sequencing data from TCGA cohort, *TNFAIP2* expression was significantly correlated with OS of patients in nine cancer types (Fig. 3a-i). Upregulation of *TNFAIP2* was associated with a favorable OS in BLCA, SARC, SKCM and STAD patients. In contrast, high level of *TNFAIP2* expression was associated with inferior OS in AML, KIRC, UVM, LGG and THYM patients.

### *TNFAIP2* was an independent prognostic factor for AML patients

**Fig. 4:**
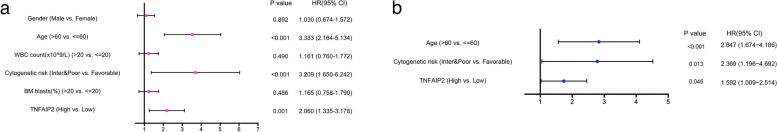
*TNFAIP2* overexpression as an independent prognostic factor in AML patients. **a** Forest plot for univariate cox regression analysis of *TNFAIP2* mRNA expression with OS in AML with different clinicopathological features. **b** Forest plot for multivariate cox regression analysis of *TNFAIP2* mRNA expression with OS in AML with different clinicopathological features

As Kaplan-Meier curves and log-rank test analyses indicated that AML patients with high *TNFAIP2* expression had significantly inferior OS than patients with low *TNFAIP2* expression in Timer 2.0 database, cox proportional-hazards model was used to confirm the potential of *TNFAIP2* as a prognostic factor in AML patients from TCGA dataset. Univariate cox regression suggested that *TNFAIP2* expression (high vs. low, HR = 2.060, 95% CI: 1.335–3.178, *P* = 0.001), age (> 60 vs. <=60, HR = 3.333, 95% CI: 2.164–5.134, *P* < 0.001) and cytogenetic risk (intermediate & poor vs. favorable, HR = 3.209, 95% CI: 1.650–6.242, *P* < 0.001) were related to OS (Fig. 4a). Multivariate cox regression revealed that *TNFAIP2* expression (HR = 1.592, 95% CI: 1.009–2.514, *P* = 0.046), age (HR = 2.647, 95% CI: 1.674–4.186, *P* < 0.001) and cytogenetic risk (HR = 2.369, 95% CI: 1.196–4.692, *P* = 0.013) were independent prognostic factors for OS in AML (Fig. 4b).

### *TNFAIP2* mRNA expression and clinical features in AML

In view of the prognostic significance of *TNFAIP2* expression in AML and the unclear mechanism of *TNFAIP2* dysregulation in leukemogenesis, we focused on studying the biological role of *TNFAIP2* in AML. The clinical and genetic features of AML patients from the TCGA cohort were summarized in Table 1. Significant differences could be observed in the distribution of age, white blood cell (WBC) count, FAB classification, cytogenetics risk stratification, and cytogenetic alterations between *TNFAIP2*^low^ and *TNFAIP2*^high^ AML patients. Low expression of *TNFAIP2* was associated with younger disease onset age (*P* < 0.001), lower WBC count (*P* = 0.018), favorable cytogenetic risk (*P* = 0.024), t(15;17) (*P* = 0.001) and t(8;21) (*P* = 0.014). Decreased expression of *TNFAIP2* was significantly correlated with FAB-M2 (*P* = 0.006) and FAB-M3 (*P* = 0.001) while increased expression of *TNFAIP2* was correlated with FAB-M4 (*P* < 0.001) and FAB-M5 (*P* = 0.034) in the distribution of FAB classifications.

**Table 1:** Clinicopathological characteristics of AML patients from TCGA cohort

Characteristic	Low expression of ***TNFAIP2*** (***n*** = 75)	High expression of ***TNFAIP2*** (***n*** = 76)	***P*** value
Gender (male), n (%)	41 (27.2%)	42 (27.8%)	1.000
Age, median (IQR)	51 (35.5, 61.5)	62 (48, 70.25)	< 0.001
WBC count(× 10^9^/L), median (IQR)	13 (3, 39.5)	27 (6.75, 69)	0.018
Cytogenetic risk, n (%)			
Favorable	21 (14.1%)	10 (6.7%)	0.024
Intermediate	38 (25.5%)	44 (29.5%)	0.369
Poor	15 (10.1%)	21 (14.1%)	0.27
FAB classifications, n (%)			
M0	9 (6%)	6 (4%)	0.384
M1	16 (10.7%)	19 (12.7%)	0.625
M2	26 (17.3%)	12 (8%)	0.006
M3	14 (9.3%)	1 (0.7%)	0.001
M4	4 (2.7%)	25 (16.7%)	< 0.001
M5	3 (2%)	12 (8%)	0.034
M6	1 (0.7%)	1 (0.7%)	1.000
M7	1 (0.7%)	0 (0%)	0.493
Cytogenetics, n (%)			
Normal	35 (25.9%)	34 (25.2%)	0.718
+ 8	3 (2.2%)	5 (3.7%)	0.620
del(5)	1 (0.7%)	0 (0%)	1.000
del(7)	1 (0.7%)	5 (3.7%)	0.172
inv.(16)	1 (0.7%)	7 (5.2%)	0.051
t(15;17)	11 (8.1%)	0 (0%)	0.001
t(8;21)	7 (5.2%)	0 (0%)	0.014
t(9;11)	0 (0%)	1 (0.7%)	0.478
Complex	12 (8.9%)	12 (8.9%)	0.809
OS event, n (%)			0.023
Alive	34 (22.5%)	20 (13.2%)	
Dead	41 (27.2%)	56 (37.1%)	

*n* number of patients, *IQR* Interquartile range, *WBC* White blood cell, *FAB* French–American–British subtype

**Fig. 5:**
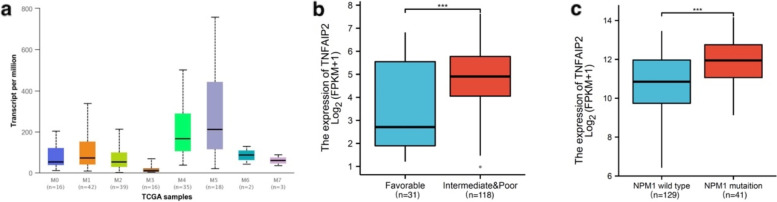
*TNFAIP2* expression and clinical features in AML patients in TCGA dataset. **a** Comparison of *TNFAIP2* expression level among different subtypes of AML in the distribution of FAB classifications analyzed by UALCAN. **b** Comparison of *TNFAIP2* expression level in AML patients according to cytogenetic risk stratification. **c** Comparison of *TNFAIP2* expression level in AML patients according to *NPM1* gene mutation status

We also analyzed the differential expression of *TNFAIP2* in AML patients from the TCGA dataset according to FAB classification and genetic alterations. As shown in Fig. 5a, *TNFAIP2* was differentially expressed among different FAB subtypes of AML with the lowest expression in FAB-M3 and highest in FAB-M4/M5 patients (Fig. 5a, Supplementary Table S1). With respect to cytogenetics risk stratification according to NCCN guideline of AML, AML patients with favorable cytogenetics risk had significantly lower expression of *TNFAIP2* than those with intermediate and unfavorable cytogenetics risk (*P* = 0.001. Fig. 5b). Moreover, *NPM1* mutation was associated with increased expression of *TNFAIP2* (*P* < 0.001. Fig. 5c).

To further validate whether *TNFAIP2* expression was linked to AML subtypes and genetic alterations, microarray data (GSE14468) from the GEO database as well as Beat AML dataset were applied to evaluate the *TNFAIP2* expression in the distribution of FAB classification, cytogenetic risk stratification and gene mutations. In GSE14468 microarray, FAB-M4/M5 patients exhibited the highest expression of *TNFAIP2* whereas FAB-M3 patients exhibited the lowest (Supplementary Fig. S3a, Supplementary Table S2). *TNFAIP2* expression level was significantly lower in AML patients with favorable cytogenetic risk compared with those with intermediate (Supplementary Fig. S3b, *P* < 0.001) or unfavorable cytogenetics risk (Supplementary Fig. S3b, *P* < 0.001). Moreover, AML patients with common genetic mutations, including *FLT3-ITD/TKD* mutation (Supplementary Fig. S3c, *P* = 0.003), *IDH1* mutation (Supplementary Fig. S3d, *P* = 0.011), *NPM1* mutation (Supplementary Fig. S3e, *P* < 0.001) or *N-RAS* mutation (Supplementary Fig. S3f, *P* < 0.001) was significantly associated with higher expression of *TNFAIP2* than those without these mutations. In contrast, AML patients with *CEBPA* double mutations had lower expression of *TNFAIP2* than wild type *CEBPA* (Supplementary Fig. S3g, *P* < 0.001) or with single *CEBPA* mutation (Supplementary Fig. S3g, *P* = 0.025). *TNFAIP2* expression was also significantly increased in patients with *EVI* expression in comparison to those without *EVI* expression (Supplementary Fig. S3h, *P* = 0.005). On the other hand, the expression pattens of *TNFAIP2* in the distribution of FAB classification (Supplementary Fig. S3i) and *NPM1* gene mutations (Supplementary Fig. S3j) in Beat AML dataset was similar to those in GSE14468 microarray.

### *TNFAIP2*-coexpressed gene analysis in AML patients

**Fig. 6:**
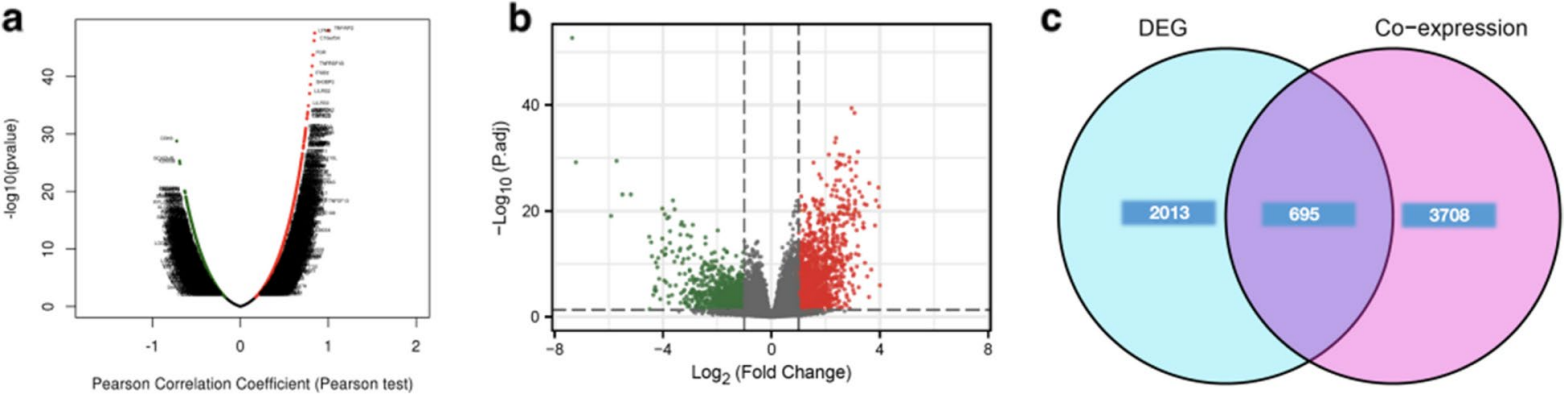
Genome-wide genes associated with *TNFAIP2* expression in AML. **a** Volcano plot for the co-expressed genes associated with *TNFAIP2* expression, analyzed by LinkedOmics. **b** Volcano plot for differentially expressed genes (DEGs) between *TNFAIP2*^high^ and *TNFAIP2*^low^ groups. **c** Venn diagram for the overlapping genes between significantly co-expressed genes and significantly DEGs of *TNFAIP2*

To further investigate the mechanism of *TNFAIP2* in leukemogenesis, the co-expressed genes in conjunction with *TNFAIP2* in AML patients in the TCGA dataset were firstly investigated by LinkedOmics (Fig. 6a). The results indicated that a total of 4403 co-expressed genes were significantly correlated with *TNFAIP2* in AML (FDR < 0.05, *P* < 0.05, and |cor.| ≥ 0.3, Supplementary Table S3). Among the 4403 genes, 1899 amongst were positively correlated with *TNFAIP2* expression whereas 2504 were negatively correlated with *TNFAIP2*.

Subsequently, the DEGs between *TNFAIP2*^high^ and *TNFAIP2*^low^ groups were also compared in AML. As shown in Fig. 6b and Supplementary Table S4, a total of 2708 DEGs were identified (*P* < 0.05, |log2 FC| ≥ 1), of which 1460 upregulated genes and 1248 downregulated genes represented were detected in *TNFAIP2*^high^ group, respectively.

When comparing these 2708 DEGs of *TNFAIP2* with the 4403 significantly co-expressed genes aforementioned by Draw Venn diagrams online tool, 695 overlapping genes, consisting of 538 positively upregulated genes and 157 negatively downregulated genes (Fig. 6c, Supplementary Table S5), were identified for further functional analysis.

### Functional enrichment analysis of *TNFAIP2* in AML

**Fig. 7:**
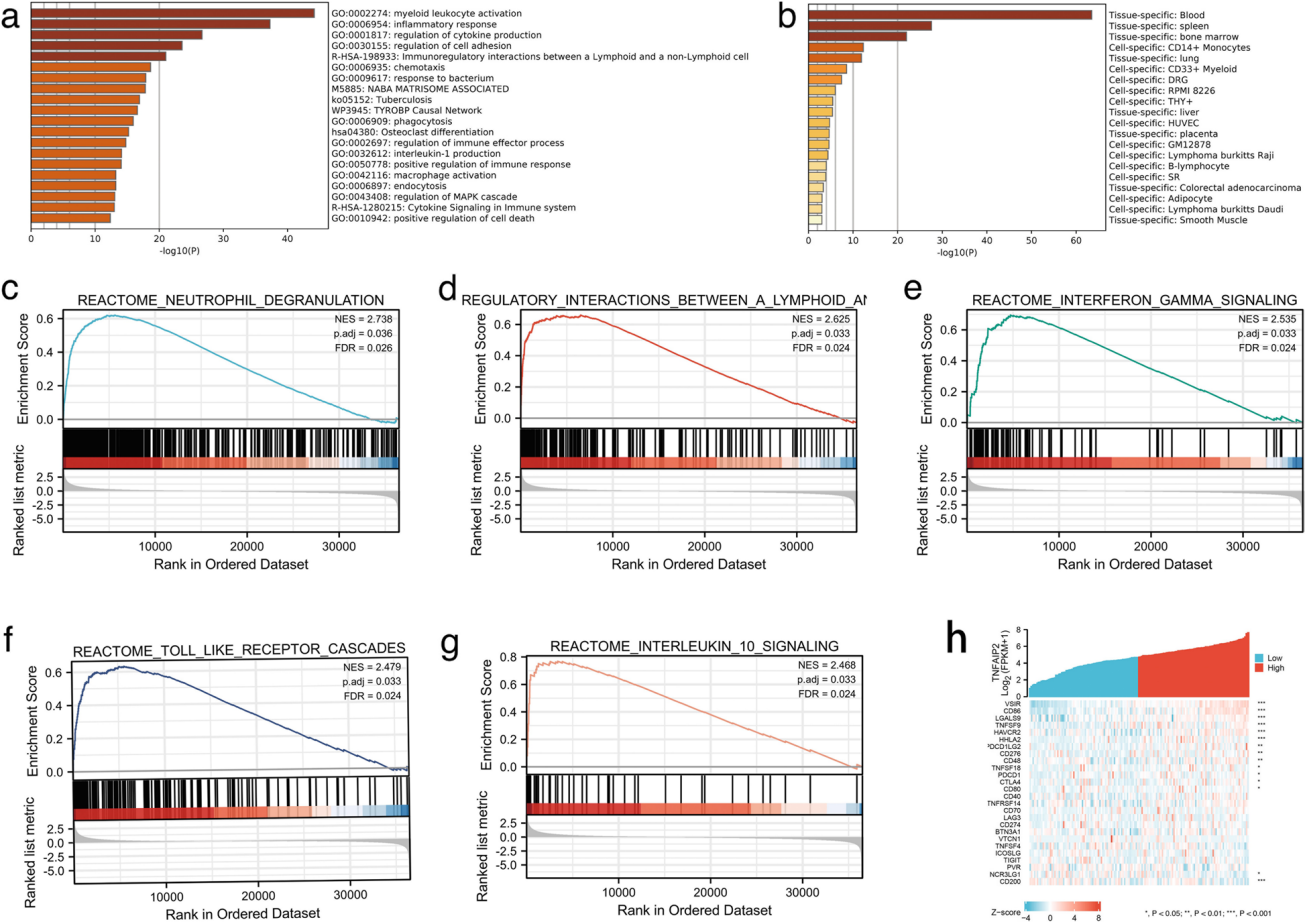
Functional enrichment analysis of overlapping genes in AML. **a** Enrichment analysis of GO and KEGG pathway associated with *TNFAIP2* expression analyzed by Metascape; **b** Enrichment analysis of overlapping genes in tissues and cells analyzed by Metascape; **c-g** Signaling pathways enriched by GSEA analyses of overlapping genes. **h** Co-expression of *TNFAIP2* and immune-related genes in AML

To investigate the biological function of 695 overlapping genes, GO/KEGG analyses were performed by Metascape database. The top 20 of enriched sets were listed in Fig. 7a. The enrichment analysis results suggested that *TNFAIP2* and its-related partners were functional mediators for immunological modulation, including myeloid leukocyte activation, immunoregulatory interactions between a lymphoid and a non-lymphoid cell, regulation of cell adhesion, regulation of cytokine production, regulation of immune effector process, interleukin-1 production, positive regulation of immune response and cytokine signaling in immune system. These genes were also linked to inflammation process, including response to bacterium, tuberculosis, phagocytosis, macrophage activation and endocytosis. In addition, *TNFAIP2* expression was associated with regulation of MAPK cascade and cell death. On the other hand, as demonstrated in Fig. 7b, the overlapping genes were enriched in blood, spleen and bone marrow (CD14+ monocytes and CD33+ myeloid cells), providing additional evidence of immunomodulatory role of *TNFAIP2* in the pathogenesis of AML.

To further explore the molecular pathways that were significantly altered in AML between *TNFAIP2*^high^ and *TNFAIP2*^low^ groups, GSEA analyses were conducted using the GSEA software. The data indicated that *TNFAIP2* mainly positively regulated immune-related processes or pathways, including neutrophil degranulation (Fig. 7c), immunoregulatory interactions between a lymphoid and a non-lymphoid cell (Fig. 7d), interferon-gamma signaling (Fig. 7e), Toll-like receptor cascades (Fig. 7f), and interleukin-10 signaling (Fig. 7g), etc., further suggesting the immunological function of *TNFAIP2* in leukemogenesis.

The enrichment analysis indicated that *TNFAIP2* participated immune network in AML, therefore, gene co-expression analyses were performed to explore the correlation between *TNFAIP2* expression and immune-related genes in AML patients. The analyzed genes encoded immune checkpoint. As shown in Fig. 7h, *TNFAIP2* expression was significantly linked to nearly half of immune checkpoint- related genes expressions, including *VSIR*, *CD86*, *LGALS9*, *CD200,* etc.

### PPI networks of *TNFAIP2*-related partners

**Fig. 8:**
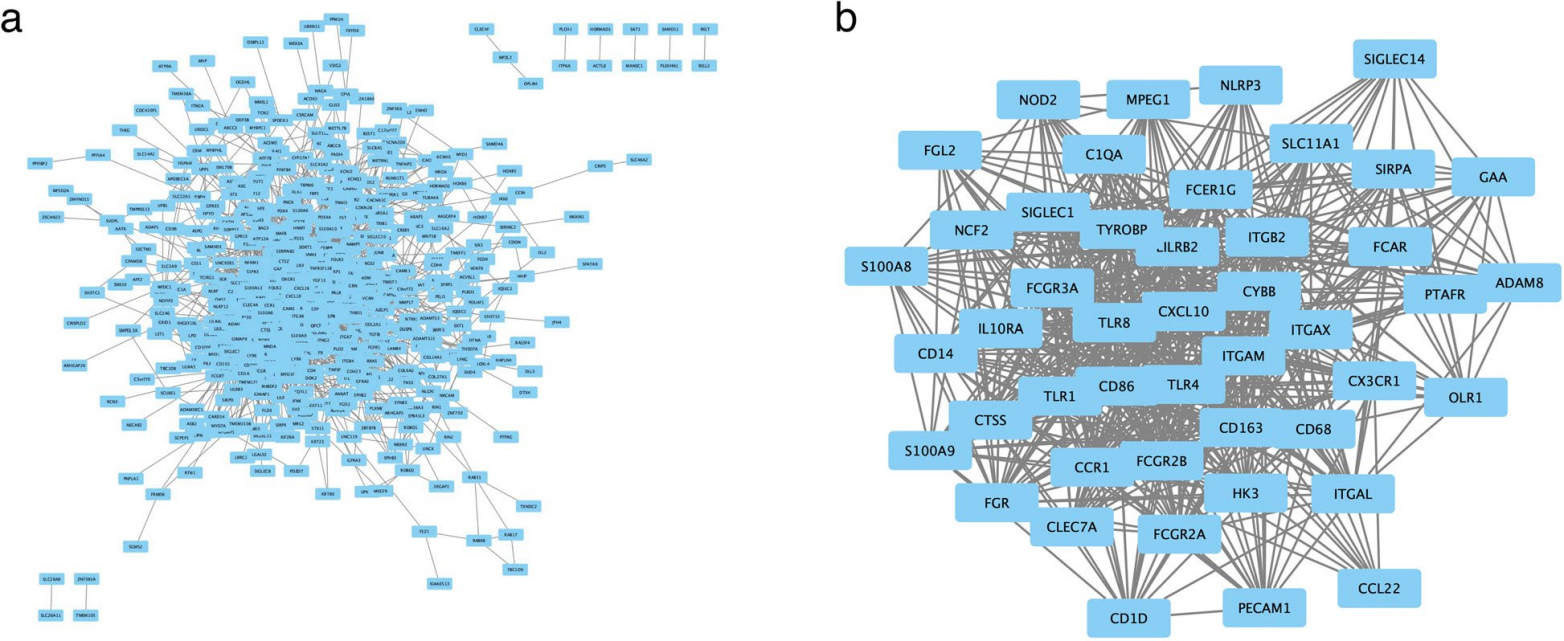
Protein-protein interaction (PPI) network of overlapping genes in AML. **a** PPI network for the overlapping genes, analyzed by String; **b** Network of hub genes screened from overlapping genes by MCODE analysis with Cytoscape software

PPI network of overlapping genes were analyzed by using String. The figure of PPI network visualized by Cytoscape software was shown in (Fig. 8a). Upon inputting the 695 overlapping genes plus *TNFAIP2*, a total of 606 nodes and 4394 edges was obtained. Subsequently, PPI network was further analyzed by using the MCODE plugin in Cytoscape software to screen for hub genes. The results of MCODE analysis indicated that the most significant module (MCODE score = 25.689) consisted of 46 hub genes (Fig. 8b) which were all upregulated in AML. Among these hub genes, 13 genes, including *NCF2*, *FGR*, *ITGAL*, *CXCL10*, *LILRB2, FCGR2B*, *PECAM1*, *CD163*, *ITGAM, SIGLEC1, ITGAX*, *ITGB2* and *S100A8*, were significantly associated with inferior OS of AML patients (Supplementary Fig. S4).

### Oncogenic function on *TNFAIP2* in AML cells

**Fig. 9:**
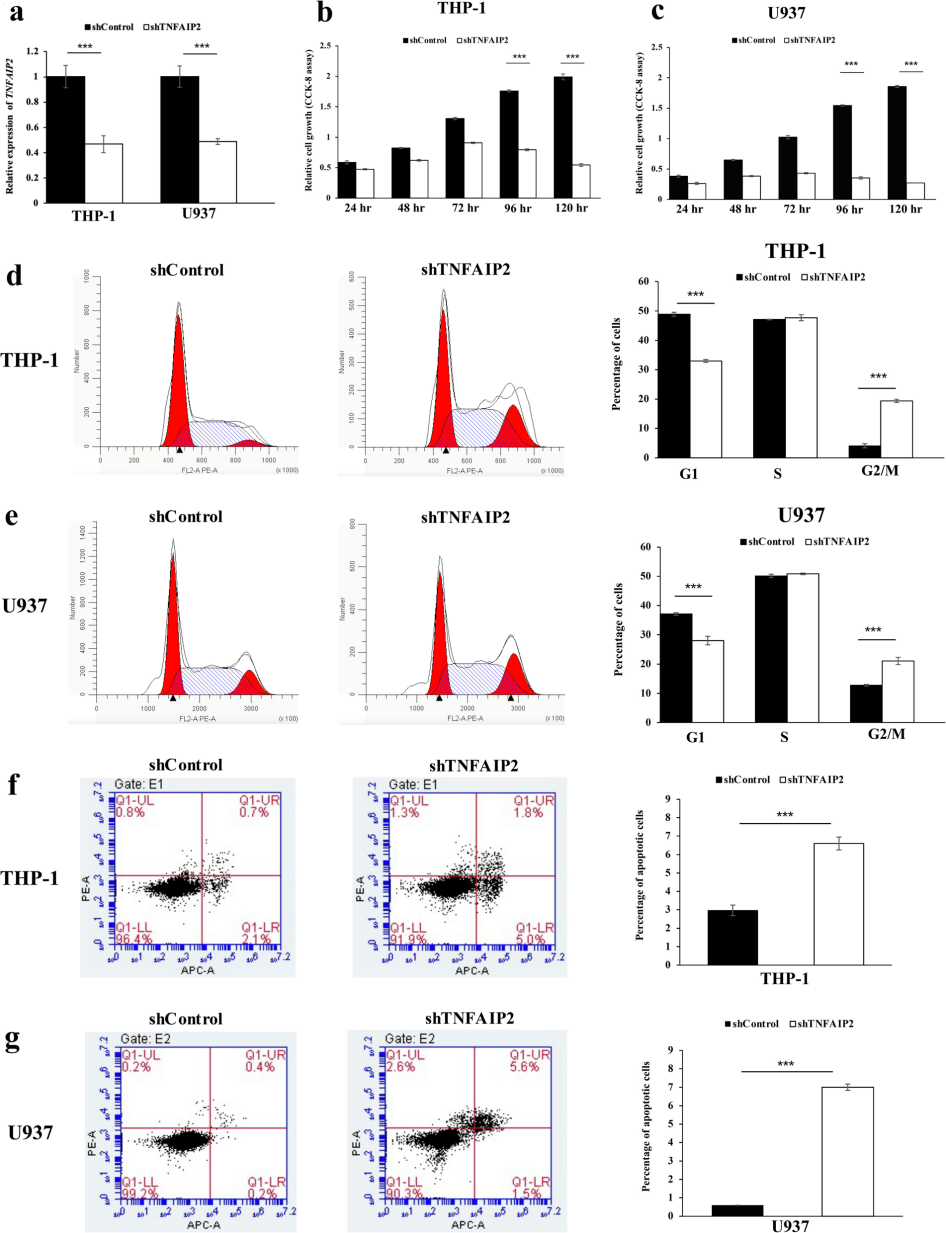
Oncogenic function of *TNFAIP2* in AML cells. **a** qRT-PCR was performed to examine the efficiency of *TNFAIP2* silencing in short hairpin RNA-stably transduced AML cell lines THP-1 and U937 at 120-hour post-transfection. **b, c** Cellular proliferation upon silencing of *TNFAIP2* in THP-1 (**b**) and U937 (**c**) cells was studied by CCK-8 assay after transfection. **d**,** e** The cell-cycle distribution upon silencing of *TNFAIP2* in THP-1 (**d**) and U937 (**e**) cells was studied by flow cytometry at 120-hour post-transfection. **f**,** g** The cell apoptosis upon silencing of *TNFAIP2* in THP-1 (**f**) and U937 (**g**) cells was studied by flow cytometry at 120-hour post-transfection. Columns represented mean +/− 1SD from three independent experiments

Because *TNFAIP2* expression was upregulated in AML patient samples and associated with poor survivals, we explored the biological role of *TNFAIP2* in AML by establishing stable *TNFAIP2* knockdown AML cell lines THP-1 and U937 cell lines via lentivirus infection. qRT-qPCR was used to verify the effectiveness of shRNA knockdown. The levels of *TNFAIP2* mRNAs were obviously decreased in THP-1 and U937 cells after transfection with *TNFAIP2* shRNA in comparison with the scramble control cells (Fig. 9a).

CCK-8 assays were performed to measure the proliferation of THP-1 and U937 cells subjected to knockdown. The results revealed that depletion of *TNFAIP2* significantly reduced cell proliferation compared with scramble control in both cell lines (Fig. 9b, c). To further explore the growth inhibition observed following *TNFAIP2* knockdown, the cell-cycle profiles of *TNFAIP2* knockdown cells were compared with scramble controls by flow cytometry. Suppression of *TNFAIP2* resulted in a decrease in the number of cells in the G1-phase and an increase in the percentage of cells in the G2/M phase (Fig. 9d, e). Furthermore, the effect of depletion of *TNFAIP2* on cell apoptosis was studied by Annexin V-APC & PI assay. A flow cytometry analysis demonstrated that both THP-1 and U937 cells exhibited enhanced early and late apoptosis under *TNFAIP2* suppression compared with the scramble control groups (Figs. 9f, g). Taken together, these results indicated the oncogenic role of *TNFAIP2* in AML cells via inhibiting cellular proliferation, cell cycle arrest and inducing cell apoptosis.

## Discussion

Numerous reports showed that *TNFAIP2* functioned as a mediator for inflammation, angiogenesis, cell proliferation and hematopoiesis [11, 16, 17, 19, 20, 42, 43]. Dysregulation of *TNFAIP2* was implicated in infectious diseases and cancers [11, 16, 22, 23, 43, 44]. However, there have been few studies about *TNFAIP2* in leukemogenesis [45]. Hence, the mRNA expression and prognostic values of *TNFAIP2* was comprehensively explored in pan-cancers by utilizing public datasets in this study. Furthermore, the clinical significance and role of *TNFAIP2* in AML were also investigated by bioinformatic analysis and functional assays for the first time. Several observations were made in this study.

Firstly, the role of *TNFAIP2* in carcinogenesis may vary in different cancers. Although *TNFAIP2* expression was reported upregulated in several types of cancers, including breast cancers, glioma, and nasopharyngeal carcinoma [16, 22, 23, 43], the current study demonstrated that *TNFAIP2* was also found to be downregulated in different types of tumors including adrenocortical carcinoma, kidney renal clear cell carcinoma, prostate adenocarcinoma, etc., indicating that *TNFAIP2* was expressed in a tissue-specific manner. However, Jia et al. showed that *TNFAIP2* expression was increased in breast cancer tissues [43], which contradicted with our current results, possibly due to the difference in histological subtypes of tumor studied. On the other hand, prognostic value of *TNFAIP2* expression in cancer patients has been reported rarely so far [46]. The present study showed multifaceted prognostic effect of *TNFAIP2* overexpression on OS in certain types of cancers based on TCGA and GEO database, supported by an association with high expression of *TNFAIP2* and better OS in STAD, BLCA, SARC, and SKCM whereas inferior OS in AML, KIRC, UVM, LGG, THYM，DLBCL, meningioma and lung adenocarcinoma. Taken together, these data suggested that the expression level and prognostic significance of *TNFAIP2* was highly cancer-dependent, which needs to be further confirmed for the specific role of *TNFAIP2* in each cancer.

Rusiniak E, et al. previously reported that *TNFAIP2* was downregulated in acute promyelocytic leukemia (APL) and could be induced by retinoic acid (RA) in PML-RARα-positive cells, suggesting that *TNFAIP2* was involved in RA signaling in APL [45]. The role of *TNFAIP2* in other subtypes of AML has not been well studied so far. In our research, expression level of *TNFAIP2* was significantly increased in AML. Moreover, AML patients with *TNFAIP2* overexpression demonstrated shorter OS in TCGA datasets. Analyses of clinical parameters of AML patients from the TCGA cohort also showed that upregulation of *TNFAIP2* was an independent novel prognostic biomarker for OS in AML. In addition, the relations between *TNFAIP2* expression and clinical or genetic phenotypes of AML patients were further examined. The results suggested that APL patients (FAB-M3) had the lowest expression of *TNFAIP2* while monocytic subtypes of AML patients (FAB-M4/M5) had the highest of *TNFAIP2*. Importantly, *TNFAIP2* overexpression was associated with unfavorable cytogenetic risk and AML-related gene mutations, including *FLT3*-ITD mutation, *FLT3*-TKD mutation, *IDH1* mutation, *NPM1* mutation and *N-RAS* mutation. Overall, these data implicated the critical role of *TNFAIP2* in the pathogenesis of AML.

Furthermore, we analyzed the genetic and epigenetic alterations of *TNFAIP2* in AML. None of genetic alterations, such as gene mutation, deletion or amplification were found. As for DNA methylation, we observed an obvious association between increased DNA methylation level at gene-body region and high expression of *TNFAIP2*. Nevertheless, there was no correlation between promoter DNA methylation and mRNA level of *TNFAIP2*. In general, cancer cells are characterized by two major alterations of DNA methylation: global DNA hypomethylation while gene-specific DNA hypermethylation of promoter-associated CpG island. Moreover, promoter DNA hypermethylation could mediate reversible silencing of tumor suppressor genes. The role of gene body methylation, i.e., the methylation of CpG sites throughout the introns and exons, in carcinogenesis is far more unclear. Some studies implicated that high methylation level at gene body is correlated with high gene expression [47, 48], which was consistent with our results. Nevertheless, the concrete mechanisms have not yet been elucidated. Numerous studies indicated that DNA methylation showed impact on the prognosis in different cancers [49–51]. Our study also demonstrated the correlation of hypermethylation of *TNFAIP2* at gene body region with poor OS in AML patients, which might be explained by the impact of high expression of *TNFAIP2* caused by gene body hypermethylation on adverse OS. Therefore, in addition to *TNFAIP2* expression, *TNFAIP2* methylation at gene body could also be considered as a potential prognostic biomarker in AML.

In the current study, the biological role of *TNFAIP2* in AML was explored by functional assay via lentivirus transduction. In AML cells THP-1 and U937, knockdown *TNFAIP2* led to inhibition of cellar inhibition, cell cycle arrest and increase of apoptosis, indicated the oncogenic function of *TNFAIP2* in AML, which was consistent in other type of cancers [11, 16, 21–25]. Moreover, the function and mechanism of *TNFAIP2* in leukemogenesis was predicted by enrichment analysis. *TNFAIP2* was previously reported to be abundant in immune cells such as myelomonocytic cells, endothelial cells, peripheral blood monocytes, dendritic cells, macrophages, etc., and is implicated in immune response in the process of septic shock [16, 52]. As expected, the current GO/KEGG and GSEA analysis indicated for the first time that the function of *TNFAIP2* in leukemogenesis was primarily related to positive regulation of immune response, such as myeloid leukocyte activation, neutrophil degranulation, immunoregulatory interactions between a lymphoid and a non-lymphoid cell, regulation of cell adhesion, regulation of cytokines production and signaling (interleukin-1, interleukin-10, interferon-gamma) and so on. Moreover, PPI network of *TNFAIP2*-related partners were constructed and ultimately 46 hub genes were screened. Among these hub genes, 13 genes had prognostic impact on OS in patients with AML.

## Conclusion

*TNFAIP2* was expressed across different tissues in a tissue-specific manner. Upregulation of *TNFAIP2* was found in AML, particularly in FAB-M4/5 patients. Furthermore, our study suggested *TNFAIP2* as a novel prognostic predictor and its relationship with cytogenetic risk stratification and disease-related gene mutations in AML patients. Hypermethylation of *TNFAIP2* at gene body was associated with upregulation of *TNFAIP2* and inferior OS in AML. Functional enrichment analysis suggested the involvement of immunoregulation of *TNFAIP2* in the occurrence and development of AML. Further in vitro studies are warranted to functionally validate the significance of *TNFAIP2* in AML tumor immunology.

## Supplementary Information

**Additional file 1: Supplementary Table S1.** Statistical results of Student’s T-test for *TNFAIP2* expression level among different subtypes of AML patients from TCGA cohort in the distribution of FAB classifications. The results were analyzed by UALCAN. **Supplementary Table S2.** Statistical results of Student’s T-test for *TNFAIP2* expression level among different subtypes of AML patients in the microarray data of GSE14468 from the GEO database in the distribution of FAB classifications. **Supplementary Table S3.** The co-expressed genes associated with *TNFAIP2* expression in AML. **Supplementary Table S4.** The differentially expressed genes between the *TNFAIP2*^high^ and *TNFAIP2*^low^ group of AML patients. **Supplementary Table S5.** The overlapping genes between significantly co-expressed genes and significantly differentially expressed genes of *TNFAIP2* in AML.

**Additional file 2: Supplementary Fig. S1.** *TNFAIP2* expression in normal tissues and cancer cell lines. (a) *TNFAIP2* expression in normal tissues, analyzed by GTEx portal; (b) *TNFAIP2* expression in cancer cell lines analyzed by CCLE.

**Additional file 3: Supplementary Fig. S2.** Epigenetic alterations of *TNFAIP2* in AML. **(a)** The exact location of CpG sites in gene body of *TNFAIP2* from UCSC database. **(b)** Kaplan-Meier analysis of the prognostic value of *TNFAIP2* methylation at gene body region on OS in AML patients, analyzed by MethSurv.

**Additional file 4: Supplementary Fig. S3.** *TNFAIP2* expression and clinical features of AML patients in the microarray data of GSE14468 from GEO database and Beat AML dataset. (a) Comparison of *TNFAIP2* expression level among different subtypes of AML in the distribution of FAB classifications in the microarray data of GSE1446. (b) Comparison of *TNFAIP2* expression level in AML patients according to cytogenetic risk stratification in the microarray data of GSE1446. (c-h) Comparison of *TNFAIP2* expression level in AML patients according to *FLT3* mutation (c), *IDH1* mutation (d), *NPM1* mutation (e), *N-RAS* mutation (f), *CEBPA* mutation (g) and *EVI* expression (h) in the microarray data of GSE1446. (i) Comparison of *TNFAIP2* expression level among different subtypes of AML in the distribution of FAB classifications in Beat AML dataset. (j) Comparison of *TNFAIP2* expression level in AML patients according to *NPM1* mutation in Beat AML dataset.

**Additional file 5: Supplementary Fig. S4.** Prognostic value of hub genes for OS in AML patients.

## Data Availability

The datasets obtained from web-based sources and subsequently analyzed in our study were: The Cancer Genome Atlas (TCGA) database, UCSC Xena (https://xena.ucsc.edu/), CCLE database (https://portals.broadinstitute.org/ccle), Timer 2.0 database (http://timer.comp-genomics.org), cBioPortal web database (https://www.cbioportal.org/), Shiny Methylation Analysis Resource Tool (SMART) App (http://www.bioinfo-zs.com/smartapp/) database, and MethSurv database (https://biit.cs.ut.ee/methsurv/). Gene expression profile of GSE14468 dataset from Gene Expression Omnibus (GEO) database (http://www.ncbi.nlm.nih.gov/geo/).

## Abbreviations

ACC: Adrenalcortical carcinoma
AML: Acute myeloid leukemia
APL: Acute promyelocytic leukemia
BLCA: Bladder urothelial carcinoma
BRCA: Breast invasive carcinoma
CCLE: Cancer Cell Line Encyclopedia
CHOL: Cholangiocarcinoma
COAD: Colon adenocarcinoma
DFI: Disease-free interval
DLBCL: Diffuse large B-cell lymphoma
ESCA: Esophageal carcinoma
FAB: French–American–British
FDR: False discovery rate
GBM: Glioblastoma multiforme
GEO: Gene Expression Omnibus
GO: Gene Ontology
GSEA: Gene Set Enrichment Analyses
GTEx: The Genotype-Tissue Expression
HNSC: Head and Neck squamous cell carcinoma
KEGG: Kyoto Encyclopedia of Genes and Genomes
KICH: Kidney chromophobe
KIRC: Kidney renal clear cell carcinoma
KIRP: Kidney renal papillary cell carcinoma
LGG: Brain lower grade glioma
LIHC: Liver hepatocellular carcinoma
LUAD: Lung adenocarcinoma
LUSC: Lung squamous cell carcinoma
MESO: Mesothelioma
MSI: Microsatellite instability
NES: Normalized enrichment score
OS: Overall survival
OV: Ovarian serous cystadenocarcinoma
PAAD: Pancreatic adenocarcinoma
PCPG: Pheochromocytoma and paraganglioma
PPI: Protein-protein interaction
PRAD: Prostate adenocarcinoma
RA: Retinoic acid
READ: Rectum adenocarcinoma
ROC: Receive Operating Characteristic
SARC: Sarcoma
SKCM: Skin cutaneous melanoma
SMART: Shiny Methylation Analysis Resource Tool
STAD: Stomach adenocarcinoma
TCGA: The Cancer Genome Atlas
TGCT: Testicular germ cell tumor
THCA: Thyroid carcinoma
THYM: Thymoma
TNFAIP2: Tumor necrosis factor alpha-induced protein 2
UCEC: Uterine corpus endometrial carcinoma
UVM: Uveal melanoma
WBC: White blood cell
