# Supplementary figures and images for: Pan-cancer analysis of oncogenic TNFAIP2 identifying its prognostic value and immunological function in acute myeloid leukemia
Source: BMC Cancer. 2022 Oct 15;22:1068. doi: 10.1186/s12885-022-10155-9 (PMC9571470; doi:10.1186/s12885-022-10155-9)

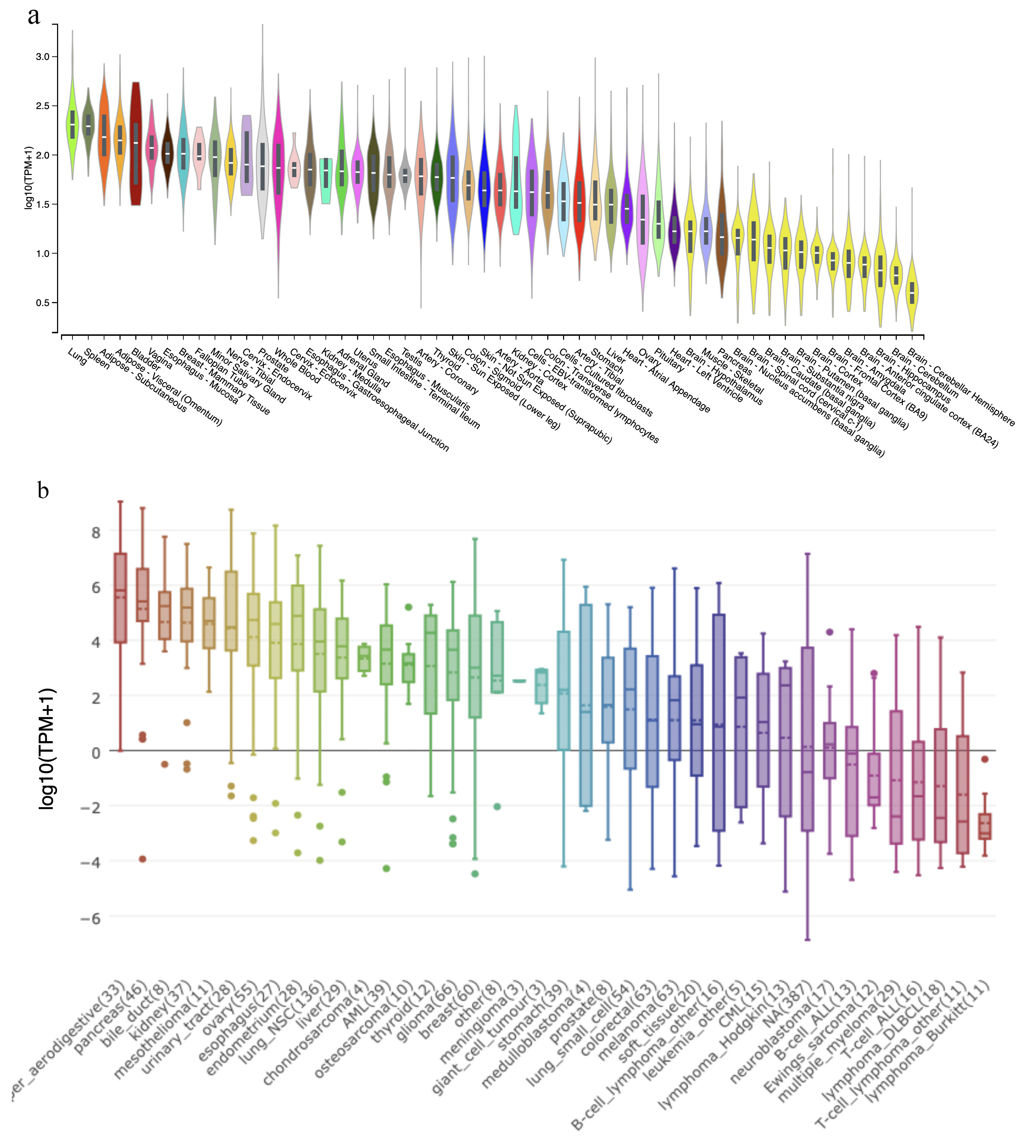

Supplement: Supplementary file 2 — Additional file 2: Supplementary Fig. S1. TNFAIP2 expression in normal tissues and cancer cell lines. (a) TNFAIP2 expression in normal tissues, analyzed by GTEx portal; (b) TNFAIP2 expression in cancer cell lines analyzed by CCLE. [file 12885_2022_10155_MOESM2_ESM.jpg]

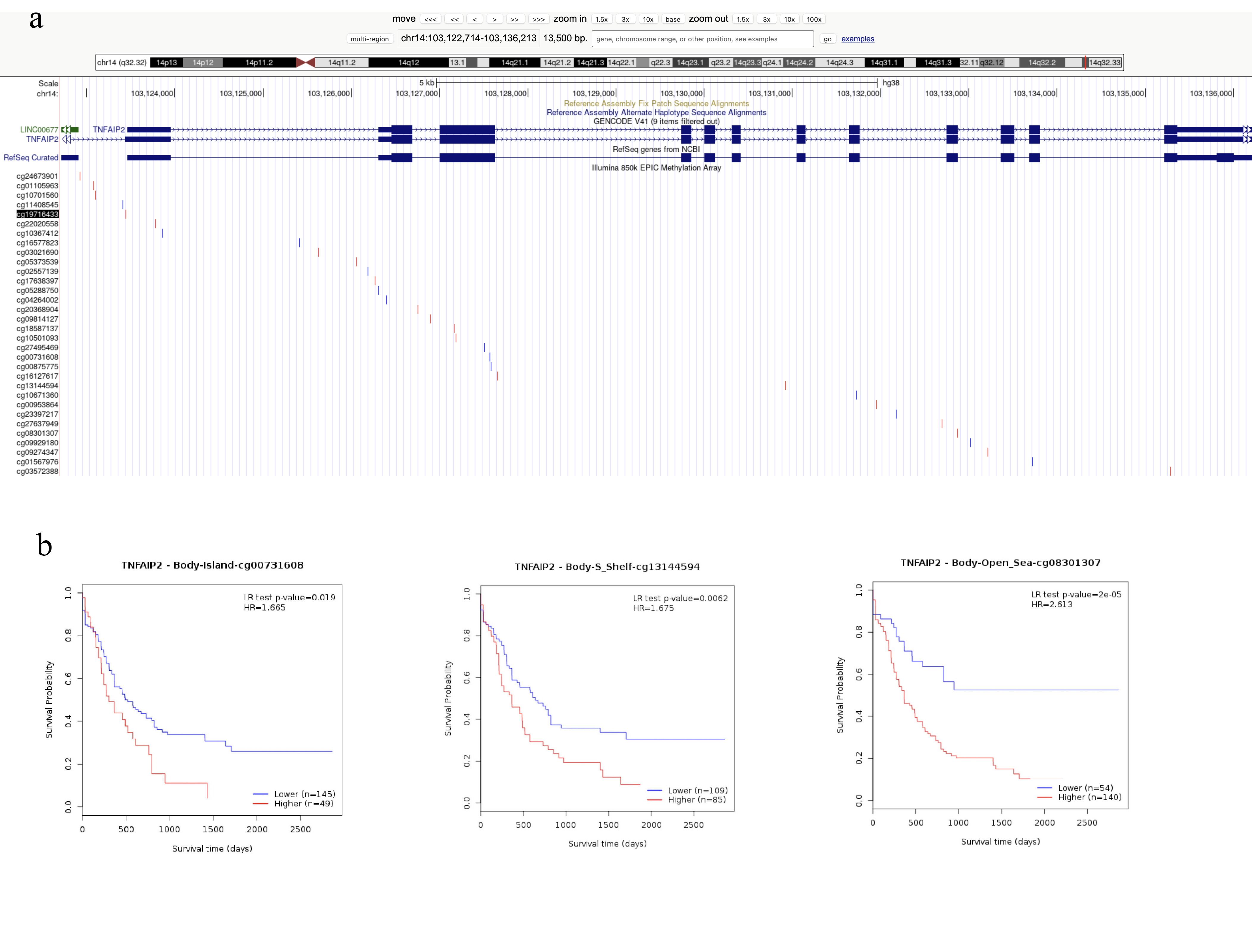

Supplement: Supplementary file 3 — Additional file 3: Supplementary Fig. S2. Epigenetic alterations of TNFAIP2 in AML. (a) The exact location of CpG sites in gene body of TNFAIP2 from UCSC database. (b) Kaplan-Meier analysis of the prognostic value of TNFAIP2 methylation at gene body region on OS in AML patients, analyzed by MethSurv. [file 12885_2022_10155_MOESM3_ESM.jpg]

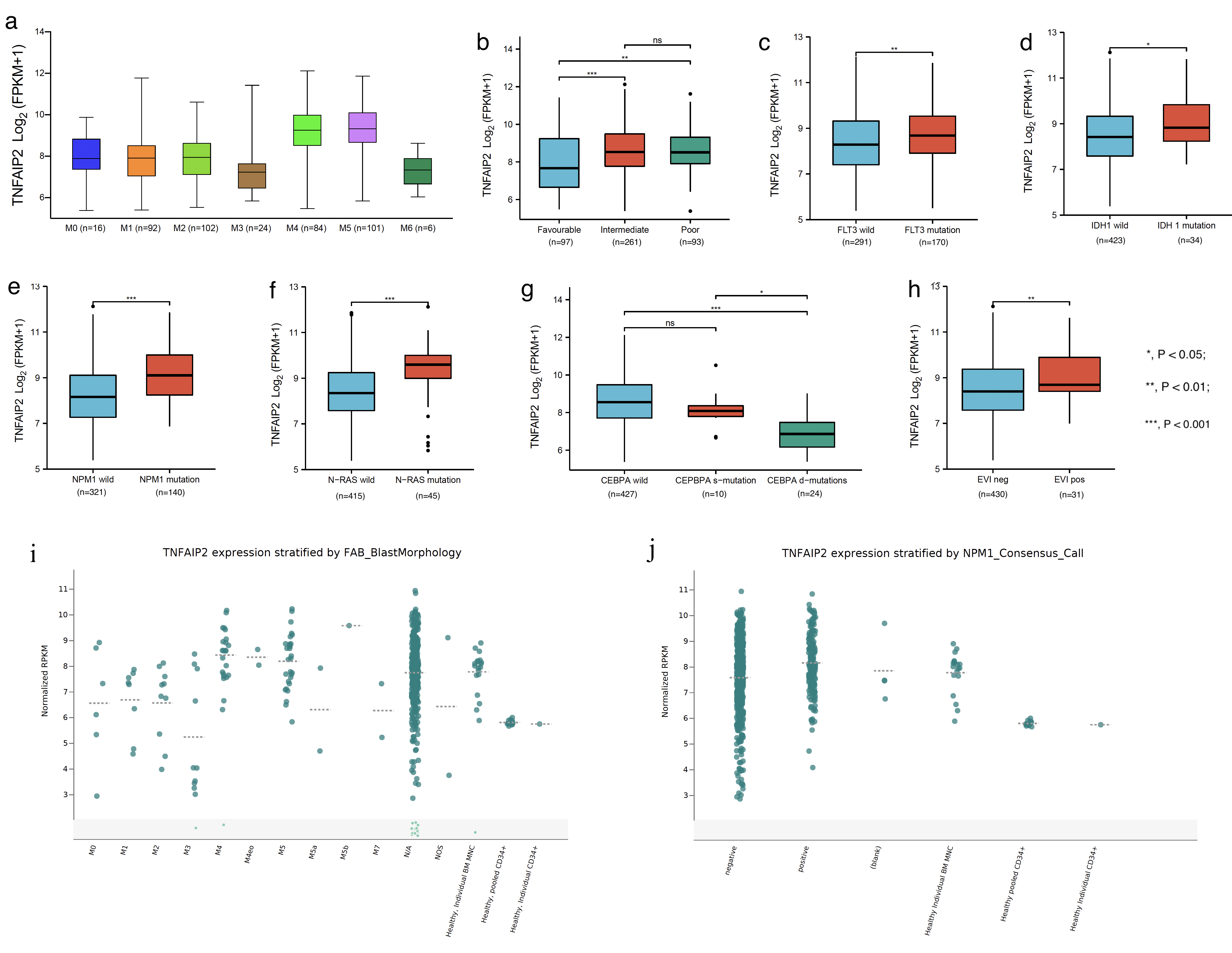

Supplement: Supplementary file 4 — Additional file 4: Supplementary Fig. S3. TNFAIP2 expression and clinical features of AML patients in the microarray data of GSE14468 from GEO database and Beat AML dataset. (a) Comparison of TNFAIP2 expression level among different subtypes of AML in the distribution of FAB classifications in the microarray data of GSE1446. (b) Comparison of TNFAIP2 expression level in AML patients according to cytogenetic risk stratification in the microarray data of GSE1446. (c-h) Comparison of TNFAIP2 expression level in AML patients according to FLT3 mutation (c), IDH1 mutation (d), NPM1 mutation (e), N-RAS mutation (f), CEBPA mutation (g) and EVI expression (h) in the microarray data of GSE1446. (i) Comparison of TNFAIP2 expression level among different subtypes of AML in the distribution of FAB classifications in Beat AML dataset. (j) Comparison of TNFAIP2 expression level in AML patients according to NPM1 mutation in Beat AML dataset. [file 12885_2022_10155_MOESM4_ESM.jpg]

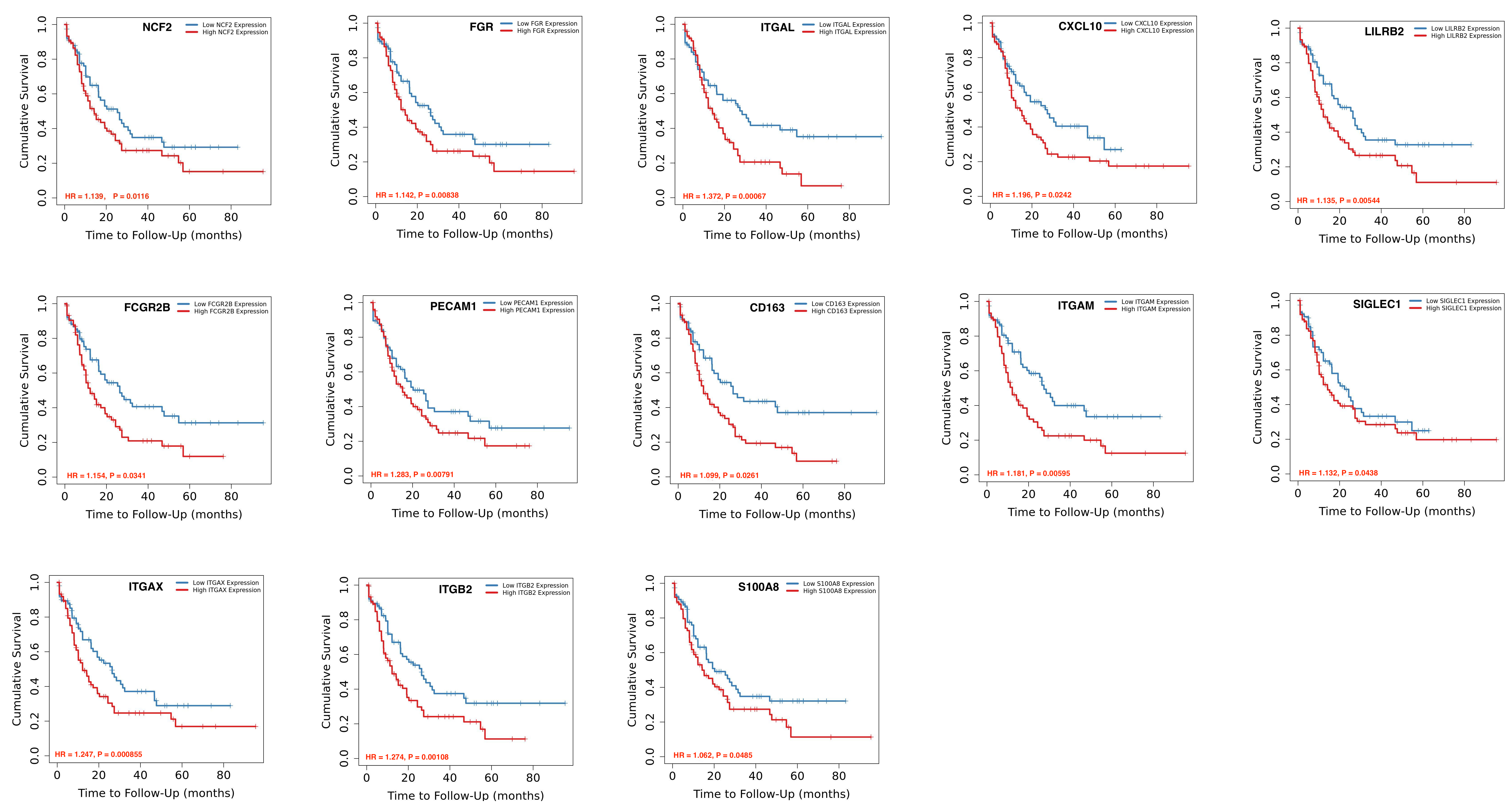

Supplement: Supplementary file 5 — Additional file 5: Supplementary Fig. S4. Prognostic value of hub genes for OS in AML patients. [file 12885_2022_10155_MOESM5_ESM.jpg]
